# Coronavirus-two infection among adults: A scoping review of literature published in 2023-24

**DOI:** 10.12669/pjms.41.6.12201

**Published:** 2025-06

**Authors:** Shehnoor Azhar, Naomi Cano Ibañez, Javier Zamora, Aurora Bueno Cavanillas

**Affiliations:** 1 Shehnoor Azhar Department of Preventive Medicine and Public Health, Faculty of Medicine, University of Granada, Spain; 2 Naomi Cano Ibañez Department of Preventive Medicine and Public Health, Faculty of Medicine, University of Granada, Spain. CIBER de Epidemiología y Salud Pública (CIBERESP-Spain), Av. Monforte de Lemos, 3-5. Pabellón 11. Planta 0 28029 Madrid, Spain Instituto de Investigación Biosanitaria de Granada (IBS.GRANADA), Av. de Madrid 15, 18012 Granada, Spain; 3 Javier Zamora Hospital Ramón y Cajal. IRYCIS. Ctra. Colmenar Viejo, Fuencarral-El Pardo, 28034 Madrid, Spain. University of Birmingham. WHO Collaborating Centre for Women’s Health Institute of Translational Medicine, Heritage Building Mindelsohn Way B15 2TH, United Kingdom. Aurora Bueno Cavanillas CIBER de Epidemiología y Salud Pública (CIBERESP-Spain), Av. Monforte de Lemos, 3-5. Pabellón 11. Planta 0 28029 Madrid, Spain; 4 Aurora Bueno Cavanillas Department of Preventive Medicine and Public Health, Faculty of Medicine, University of Granada, Spain. CIBER de Epidemiología y Salud Pública (CIBERESP-Spain), Av. Monforte de Lemos, 3-5. Pabellón 11. Planta 0 28029 Madrid, Spain Instituto de Investigación Biosanitaria de Granada (IBS.GRANADA), Av. de Madrid 15, 18012 Granada, Spain

**Keywords:** COVID-19, Low socioeconomic status, Pandemics, Self care, SARS-CoV-2

## Abstract

**Objective:**

To identify and synthesize evidence on Coronavirus-two infection (SARS-CoV-2) among adults diagnosed by polymerase chain reaction.

**Methods:**

The protocol was registered on Open Science Forum (doi: 10.17605/OSF.IO/2837X). Three bibliographic databases (Medline, SCOPUS, and Web of Science) were searched from July 2024 to December 2024. Peer-reviewed, quantitative studies with participants aged 18 and over were eligible to enlist potential risk factors of SARS-CoV-2 infection confirmed by Polymerase Chain Reaction PCR). The evidence was summarized as illustrations and tabulations with risk factors grouped into various categories. EndNote 20 was used for deduplications and organization of the literature.

**Results:**

Of 28,688 unique entries searched, 299 were shortlisted and 32 full-text manuscripts selected from 17 countries. There were two (6.2%) manuscripts based on real-time surveillance of at-risk populations. A total of 42 individual risk factors were examined in the evidence.

**Conclusion:**

Low socioeconomic status and occupation were consistent risk factors of SARS-CoV-2 infection, with minimal representation from low- and middle-income countries in the evidence body. Future research should prioritize standardized methods and inclusion of underrepresented regions to enhance global applicability and inform targeted public health interventions.

## INTRODUCTION

Unlike past infectious diseases documented comprehensively over time, severe acute respiratory syndrome of novel coronavirus (SARS-CoV-2) presented newer challenges in clinical management and prevention.^1^-^3^ While various aspects of the virus were extensively reported in scientific literature to date, relatively fewer studies examined its risk factors among healthy adults.^4^ Emergence of viral lineages further underscored the need for robust research to identify risk factors, so that self-care strategies and preventive interventions remained updated, particularly for populations at heightened risk of infection.^5^,^6^

The identification of disease risk factors precedes their confirmation in a conceptual sequence commencing with exploratory studies.^7^,^8^ Later, it leads to individualized risk predictions and finally, causation. The entire investigation cycle could be resource and time-intensive, something precluded by abrupt onset of the SARS-CoV-2.^9^,^10^ Poor forecasting has resulted in wastage of resources, miscalculations, and indecisions, ranging from erroneous estimation of ICU bed utilization in New York to panic caused by doomsday scenario based on extreme value theory.^11^ Few of the major factors cited for poor quality of forecasting particularly earlier in the pandemic included inadequate epidemiological data, lack of cross-disciplinary expertise, and poor past evidence on effects of available interventions.

More recently, data from diagnostic testing and immunizations records have been made available.^12^-^16^ It improved the overall understanding of SARS-CoV-2 infection. But its interpretability and applicability were compounded by emerging viral lineages, vaccinations, re-infections, lack of standardized diagnostics, and differences of expert opinions.14,^17^-^19^ Furthermore, longstanding resource and capacity constraints limited scientific evidence from vast populations despite being considered at disproportionate risk of infection.^20^,^21^ Very recently though, at least two studies have stood out for their use of unique approaches in analyzing risk factors.^1^,^22^ They linked Polymerase Chain Reaction (PCR) testing results (positive or negative) to spatiotemporal imagery and comprehensive socioeconomic databases, respectively. These approaches indicated continuous interest in evidence-based disease prevention particularly in risk factors of SARS-CoV-2 infection among healthy adults. It could be expected that credible evidence on a previously under-reported aspect of the pandemic could now be methodically collated along with its underlying trends.

This scoping review aimed to identify and synthesize evidence on Coronavirus-two infection (SARS-CoV-2) among adults so that disease prevention and scholarship on the topic remained updated.

## METHODS

This manuscript followed the Preferred Reporting Items for Systematic reviews and Meta-Analyses extension for Scoping Reviews or PRISMA-ScR (Supplementary File-I).^23^ The protocol was registered at Open Science Forum (OSF) dated July 2, 2024, and is accessible at *osf.io/2837x* (doi: 10.17605/OSF.IO/2837X). Its development was guided by the six-stage process outlined by Arksey and O’Malley.^24^ Ethics approval was not applicable because neither human subject was recruited nor any identifiable information of participants from any study was reported.^25^

### Selection criteria:

Peer-reviewed studies of quantitative design involving individuals over 18 years of age were eligible if they analyzed primary data as observational studies and randomized controlled trials (RCTs) or secondary data as systematic reviews (SRs) on risk factors for SARS-CoV-2 infection, confirmed via PCR. Variables of initial interest were demographic including but not limited to age and biological sex), clinical status including but not limited to Body Mass Index (BMI), comorbidities like diabetes mellitus and hypertension. Manuscripts published between January 2023 to December 2024 were searched based on a set of premises as listed below.

- It allowed evidence synthesis contextualizing coronavirus infectious disease (COVID-19) mass-immunizations.^26^
- It encouraged inclusion of studies informed by experts’ consensus that developed overtime for a previously little-known virus.^20^
- More manuscripts with rigorous methodology were likely to be scanned.^1^
- The dynamic nature of pandemic (reinfections, viral lineages, seasonality) was more likely to be captured in recent publications with greater granularity of details.^27^
- It sought to generate geographically diverse evidence considering the longstanding lack of capacity of several countries to publish health data.^21^

Manuscripts recruiting individuals less than 18 years of age or hospitalized patients were excluded. However, a few exceptions were decided in prior consultation with independent reviewer (ASG) as listed below.

- If individuals below 18 years were less than 30% of the total sample.
- The inclusion of individuals below 18 years was explicitly acknowledged and justified.
- Results and findings were reported distinguished subgroups hence allowing data extraction as per stated objectives of this scoping review.
- All other aspects of inclusion criteria were met.

### Search strategy:

Three electronic databases were searched for literature namely Web of Science, SCOPUS and Medline (via PubMed). The framework of Population, Concept, and Context (PCC) was applied to identify search terminologies relating risk factors or exposures (age, sex, diabetes, hypertension, and BMI) to SARS-VoV-2 infection diagnosis on PCR as the outcome (Supplementary File-II).^28^ It was finalized following readings of several manuscripts of interests with their citation lists, and reviews of related medical subject headings (MeSH). Pilot searches were conducted during April and May 2024. All three databases were searched from July 5 2024 to December 30 2024. All search strings along with Boolean operators were scanned across titles, abstracts, author names, text words (slightly modified by individual database), during publication period of 2023-24 (Supplementary Fil-II). The search strategy related individual risk factor to SARS-CoV-2 infection (versus searching as group(s) or set(s) of variables) so that a substantially yield was found. Authors of included studies were not contacted. The search syntax for all three databases was enlisted in Supplementary File-II.

### Screening and selection:

All searches were saved and later exported to EndNote 20 for de-duplication and organization. A screening guide was applied to titles and abstracts. An independent expert (ASG) reviewed the screening guide from its inception to practical use by the investigator.^15^ If seemingly eligible, full texts were downloaded and studied. Inclusion was justified when the manuscript complied with the given inclusion criteria enumerated below.

- A clearly focused question that investigated (a) risk factor(s) of SARS-CoV-2 infection.
- Quantitative methodology.
- Confirmatory diagnosis of infection on positive PCR test.
- Eligible population.
- The source(s) and type(s) of data (primary and/or secondary data).
- Peer-reviewed and published during 2023-24.

### Data extraction:

All relevant data was extracted from selected full text manuscripts to the main worksheet (Microsoft Excel) to which nine additional tabulations were appended. Extracted details included title, authorship, country, journal, publication year, sample size, data source(s), diagnostic criteria, statistical techniques, risk factors, any pertinent observations, variable response structure, and categories.^28^ Data extraction was done as an iterative process and led by the investigator (SA). The independent expert (ASG) reviewed data extraction tool from its inception and practical use. The correspondence and online review sessions with ASG have been recorded and archived.

### Data analysis and synthesis:

Each manuscript was assigned an identification number (study ID) comprising numeric prefix from farthest to the most recent publication in ascending order ranging from 1-32, and the suffix (initials) denoting the country (where the population was recruited from). Findings were tabulated as a summary of the manuscript characteristics, analytical model, and individual variables with status of their associations with SARS-CoV-2 infection (highlighted in bold font if associated). Furthermore, the search summary was presented as a flowchart while an evidence map (using Geographical Information System software) depicted the geographical distribution of evidence by variable categories.

## RESULTS

**Fig.1:**
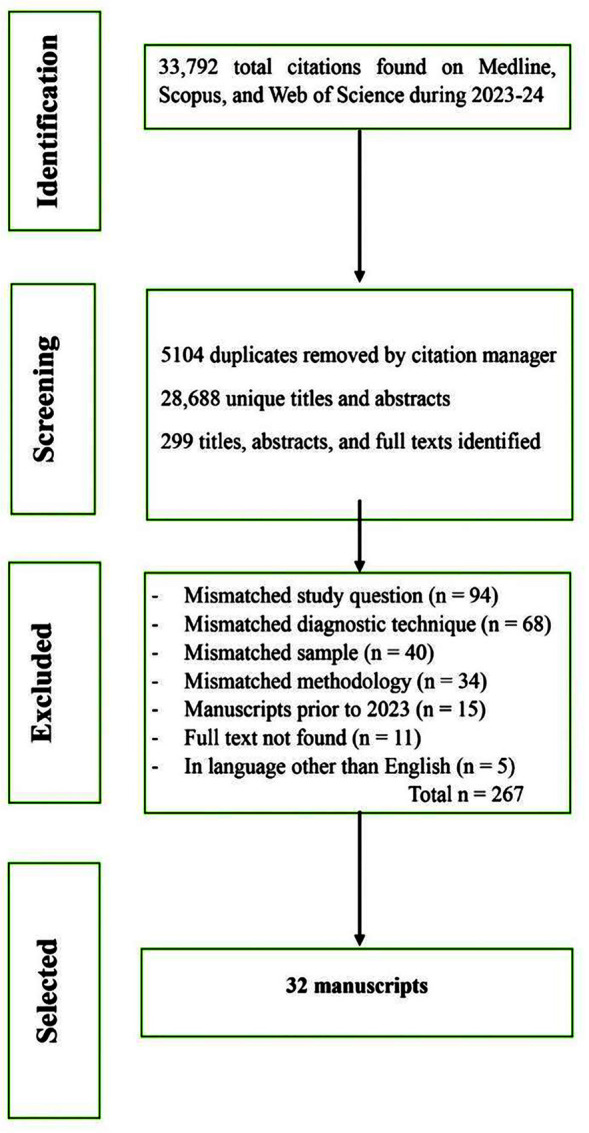
Flow diagram for the scoping review on the risk factors of SARS-CoV-2 infection among adults diagnosed by the Polymerase Chain Reaction. Illustrated under PRISMA-ScR

A total of 33,792 titles from the 2023-24 period were scanned across the three databases contributing as Medline (manuscripts n=982, 2.9%), Scopus (n=27,444, 81.2%), and Web of Science (n=5366, 15.8%). Of the 28,688 unique entries following de-duplication, 299 were shortlisted and 32 full-text manuscripts were selected as per the inclusion criteria (Fig.1). Excluded manuscripts (from 299 shortlisted) were summarized in Supplementary File-III. Fig.1: Flow diagram for the scoping review on the risk factors of SARS-CoV-2 infection among adults diagnosed by the Polymerase Chain Reaction. Illustrated under PRISMA-ScR

### Salient characteristics of synthesized evidence:

All manuscripts were based on data collected between January 2020 to March 2023^1^-^6^,^9^,^10^,^12^,^13^,^16^-^18^,^20^,^22^,^26^,^27^,^29^-^43^ and were published between February 2023 and November 2024 (data not tabulated). By study designs, 25 (78.1%) were observational^2^,^3^,^5^,^6^,^9^,^10^,^12^,^16^,^20^,^22^,^26^,^27^,^29^-^31^,^33^-^40^,^42^,^43^, 5 (15.6%) unspecified^1^,^4^,^13^,^17^,^41^ and 2 (6.2%) reported results of surveillance programs in communities.^18^,^32^ A total of 8 (25%) manuscripts identified the manufacturer of PCR diagnostic kit(s).^1^,^3^,^13^,^16^,^20^,^31^,^33^,^43^

**Fig.2:**
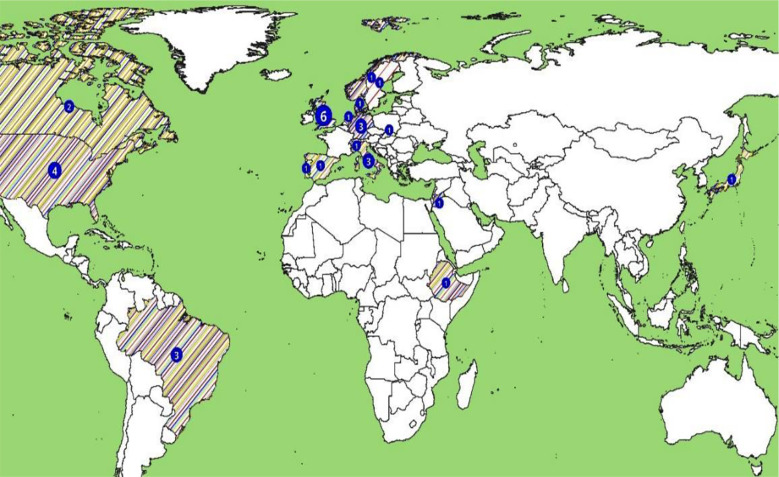
Geographic distribution of 32 manuscripts on risk factors of SARS-CoV-2 infection

The aggregated sample was 34,292,698. Among the methods used to examine associations of individual variables as possible risk factors of SARS-CoV-2 infection, multivariate regression was used as statistical technique in 28 (87.5%) of the 32 manuscripts. Of those, 18 (64.3%) used binary logistic regression. A total of 23 (71.8%) manuscripts sourced their data from more than one dataset (results not tabulated). Table-I summarized individual associations examined in each manuscript with positive relationships reported in bold font. Fig.2 (geographic distribution of evidence by risk factors’ categories) summarized the spread of evidence by countries and categories of risk factors. A total of 17 countries contributed to the selection of which 14 (82.3%) were high-income, one middle (Brazil), and two low-income (Jordan, Ethiopia). Of the total 32 manuscripts, 20 (62.5%) were European, six (18.7%) North American, 3 (9.3%) South American, 2 (6.2%) Asian, and 1 (3.1%) African. Among countries, UK published six manuscripts, USA four, Brazil, Germany, and Italy three each, Canada two, while remaining 11 countries published one each. Environmental factors were reported in a total of three manuscripts making it the least examined category.^1^,^36^,^39^

**Table-I:** Summary of individual variables (classified into six broader categories) identified in manuscripts on risk factors of SARS-CoV-2 infection among adults along with the corresponding analytical details.

Study ID	Title of the study	Variables examined (Total N = 42)					
		Demographic n* = 12	Lifestyle n = 10	Environmental n = 6	Clinical n = 6	Occupational n = 4	Viral n = 4
1-GER	Results of the Cologne Corona Surveillance (CoCoS) study – a cross-sectional study: survey data on risk factors of SARS-CoV-2 infection in adults^35^	Age, Sex, SES**	Self-care, Use public transport	---	---	Workplace mitigated risks, Job type	---
2-USA	Risk factors for COVID-19 among Californians working outside the home^41^	Age, Sex, Education, Race, SES	Use public transport	---	---	Workplace mitigated risks, Job likely to expose, Job type, Sector	---
3-USA	Community exposure among Colorado adults who tested positive for SARS-CoV-2 – A case control study, Mar – Dec 2021^40^	---	H/O socialization	---	---	Sector, Job type	---
4-ETH	SARS-CoV-2 Infections, Clinical Characteristics, and Related Risk Factors: The First 8 Months Surveillance Study Conducted in Southwest Ethiopia^3^	Age, Sex, Description of residence	H/O travel	---	DM, CLD, Symptomatic	Sector	Collection site
5-BRA	Effectiveness of a multicomponent intervention to face the COVID- 19 pandemic in Rio de Janeiro’s favelas: difference- in- differences analysis^32^	Age, Sex, Race	---	---	Current infection	Sector	Number of daily tests
6-DEN	Long-term exposure to air pollution and risk of SARS-CoV-2 infection and COVID-19 hospitalization or death: Danish nationwide cohort study^42^	Age, Sex, Education, Marital status, Nationality, SES	---	---	DM, CVD, ALRIs, Lung cancer, COPD, Dementia	---	---
7-BRA	SARS-CoV-2 Infection in Cities from the Southern Region of Bahia State, Brazil: Analysis of Variables^31^	Age, Sex, Race, Communities	---	---	DM, CKD, Symptomatic	---	---
8-UK	Ethnic inequalities in positive SARS- CoV- 2 tests, infection prognosis, COVID- 19 hospitalizations and deaths: National cohort study in Scotland^9^	Ethnicity	---	---	---	---	Dominant viral strain
9-CAN	Comparison of socio-economic determinants of COVID-19 testing and positivity in Canada: A multi-provincial analysis^30^	Age, Sex, Residence, Nationality, SES	---	---	H/O hospitalization, DM, HT	---	Number of daily tests
10-SPA	Behavioural and Personal Characteristics Associated With Risk of SARS-CoV-2 Infection in a Spanish University Cohort^29^	Age, Sex, Education, Residence, Nationality, SES	Self-care, Smoking, Use public transport, H/O socialization, Pet	---	BMI, Comorbidities	---	Collection site
11- SWE	Occupational risks associated with severe COVID-19 disease and SARS-CoV-2 infection – a Swedish national case-control study conducted from Oct 2020 to Dec 2021^6^	---	---	---	---	Workplace mitigated risks, Sector, Job likely to expose, Job type	---
12-UK	Time-sensitive testing pressures and COVID-19 outcomes: are socioeconomic inequalities over the first year of the pandemic explained by selection bias?^27^	Age, Sex, SES or SEP	---	---	---	---	Dominant viral strain, Number of daily tests
13-SLO	SARS-CoV-2 testing in the Slovak Republic from March 2020 to September 2022 – summary of the pandemic trends^16^	Age	---	---	---	---	Collection site
14-POR	Impact of sociodemographic and economic determinants of health on COVID-19 infection: incidence variation between reference periods^36^	Age, Education, Ethnicity, SES, Employment	---	H/O exposure to pollutants	---	---	Number of daily tests
15-BRA	The role of occupation in SARS-CoV-2 infection within a Brazilian municipality: A test-negative case-control study^34^	Age, Sex, Education, Race, SES	Use public transport	---	---	Occupation sector	---
16-NOR	Risk factors for SARS- CoV- 2 infection: a test- negative case–control study with population controls in Norway^38^	Age, Sex, Education, HH heating	Ventilated home and/or office, Smoking, Exercise	---	BMI, Comorbidities	---	---
17-GER	Case–control study of behavioural and societal risk factors for sporadic SARS-CoV-2 infections, Germany, 2020–2021 (CoViRiS study)^37^	SES	Self-care, Smoking, Use public transport, Ventilated home and/or office, H/O travel, socialization	---	BMI, Pulmonary diseases, Met person with flu-like symptoms	Workplace mitigated risks, Occupation sector, Job type	---
18-USA	Prevalence of SARS-CoV-2 Infection among Children and Adults in 15 US Communities, 2021^33^	Age, Sex, Race, Ethnicity	Self-care	---	Symptomatic	---	Collection site
19-GER	Results of the Cologne Corona Surveillance (CoCoS) project– a cross-sectional study^12^	Age, Sex	Smoking	---	BMI, Comorbidities	---	---
20-USA	Longitudinal Molecular and Serological Evidence of SARS-CoV-2 Infections and Vaccination Status: Community-Based Surveillance Study (CONTACT)^13^	---	---	---	Symptomatic, Onset of symptoms to testing time	Occupation sector	---
21-ITA	Keeping university open did not increase the risk of SARS-CoV-2 acquisition: A test negative case-control study among students^26^	Age, Sex, Education, Nationality, Employment	Self-care, H/O socialization, Use public transport	---	H/O exposure to a confirmed case	---	---
22-UK	Socioeconomic inequalities in risk of infection with SARS- CoV- 2 delta and omicron variants in the UK, 2020- 22: analysis of COVID- 19 Infection Survey^5^	SES	--	---	---	Occupation sector risk varied with time	Dominant viral strain
23-NET	Outdoor air pollution as a risk factor for testing positive for SARS-CoV-2: A nationwide test-negative case-control study in the Netherlands^39^	---	---	H/O exposure to PM10, PM2.5, NO_2_, Sources	---	---	---
24-JAP	Analysing factors affecting positivity in drive through COVID-19 testing: a cross-sectional study^2^	Age, Sex	H/O travel, Smoking	---	Respiratory/systemic symptoms, Time to symptoms during Omicron, H/O SARS-CoV-2, Exposure	---	Dominant viral strain, Collection site or Collector
25-UK	Changing risk factors for developing SARSCoV-2 infection from Delta to Omicron^4^	Sex, Education, Residence, Ethnicity, SES	Self-care, Disability Multigenerational HH, H/O travel, Smoking, Socialization	---	---	Workplace mitigated risks, Job likely to expose, Job type	Dominant viral strain
26-ITA	SARS-CoV-2 Positivity in Foreign-Born Adults: A Retrospective Study in Verona, Northeast Italy^20^	Age, Sex, Nationality	---	---	---	---	Sample timing, Collection site
27-SWI	Environmental and geographical factors influencing the spread of SARS-CoV-2 over 2 years: a fine-scale spatiotemporal analysis^1^	Population density, SES	---	Exposure to PM10 & PM2.5, NO_2_, Noise, Vegetation, Temperature, Coordinates (E/N)	---	---	Number of daily tests, Dominant viral strain
28-UK	Evaluating the risk of SARS-CoV-2 reinfection with the Omicron or Delta variant in Wales, UK^17^	Age, Residence	H/O Travel	---	---	---	Sample timing, Collection site
29-JOR	Risk factors for SARS-CoV-2 infection in Jordan: A cross-sectional study in the prevaccination period^43^	Age, Sex, Married, SES, Education	Handwashing, Use of disinfectant	---	Has symptoms, Comorbidity, Smoking	---	---
30-ITA	Community case study for surveillance and early case-detection of SARS-CoV-2 infections across high-risk key populations: the Sentinella programme^18^	Age, Sex	---	---	---	Occupation sector	---
31-CAN	The association of combinations of social factors and SARs-CoV-2 infection: A retrospective population-based cohort study in Ontario, 2020–2021^22^	SES	---	---	---	---	---
32-UK	Evaluation of risk- based travel policy for the COVID- 19 epidemic in Scotland: a surveillance study^10^	Age, Sex, SES	H/O travel	---	---	---	---

All associations were examined with SARS-CoV-2 as the outcome. Variables reported as associated with SARS-CoV-2 infections were highlighted in bold font.

* Each n represents the total number of unique variables within a broader category.

** Socioeconomic status (SES) was defined differently by studies - from a single measure to a compound index based on several parameters as Canadian/Scottish Index of Multiple deprivation (CIMD or SIMD).

***Abbreviations and terminologies used:*** DM (diabetes mellitus), HT (hypertension,) ALRIs (acute lower respiratory infections), CVD (coronary vessel disease),CLS (chronic liver disease), COPD (chronic obstructive pulmonary disease), BMI (body mass index), NO_2_ (nitrous oxide), BC (black carbon), NPIs (non-pharmaceutical interventions), HH (household), H/O (history of), PM (particulate matter), Job type (whether the job is predominantly Remote/hybrid/In-person/Indoor/Outdoor), Sector refers to the occupation or service sector, Collection site refers to the facility where biological specimen was obtained, Symptomatic means those of SARS-CoV-2.

### Risk factors of SARS-COV-2 infection:

A total of 42 individual variables were examined as risk factors of SARS-CoV-2 infection. Age (22 manuscripts), sex (20), socioeconomic status or SES (15), occupation (10), both education and comorbidities (9) were among the frequently examined variables. Associations of all five variables specified in search strings (age, sex, diabetes, hypertension, and BMI) remained inconclusive. Variables were grouped under six thematic categories namely demographic, lifestyle, clinical, environmental, occupational, and related to virus (Table-I). Lower SES was the risk factor (demographic) found associated with SARS-CoV-2 infection in 13 of the 15 manuscripts (86.6%), followed by smoking (lifestyle) in four of the seven manuscripts (57.1%), comorbidity (clinical) in six of the nine manuscripts (66.6%), exposure to pollutants (environmental) such as particulate matter or PM, black carbon or BC, nitrous oxide or NO_2_ in two of the three manuscripts (66.6%), occupation in seven of the 10 manuscripts (70.0%), and the pre-dominant viral lineage in 6 of the 7 manuscripts (85.7%).

### Within variable categories:

Of total 42, 12 (28.5%) variables were demographic, 10 (23.8%) lifestyle variables, 6 (14.2%) clinical variables, 6 (14.2%) environmental variables, 4 (12.5%) variables in each of the two remaining categories, occupational and related to virus. Table-I enlists all 42 variables with status of their association highlighted in bold font.

## DISCUSSION

Evidence on risk factors of SARS-CoV-2 infection has been growing gradually throughout the years 2023 and 2024 but only 32 manuscripts qualified as eligible in 28,688 unique entries. Most were observational studies based on data accumulated from various pandemic periods. Most consistently associated risk factors of SARS-CoV-2 infection (relationship examined by at least 10 manuscripts) were lower SES and occupation. Most manuscripts were European (20 manuscripts) followed by North American, (six manuscripts). All the three South American manuscripts were published by Brazil. Most manuscripts did not identify the manufacturer of PCR diagnostic kit by name.

The pandemic of SARS-CoV-2 has been a major global health event in modern history, also recorded better than any other past pandemic.^5^,^16^ Continued presentation of multifaceted evidence on the topic has remained critical for its complete control and eradication.^6^,^10^ The accumulated data could also inform professional standards and training programs to raise the workforce better skilled to handle future pandemics and health emergencies.^19^,^21^,^44^ In this context, our findings enable generation of hypotheses for credible prognostic research particularly valuable to improve health literacy among populations at heightened risks.^8^,^14^

### Strengths of findings:

This has been one of the most comprehensive and updated evidence synthesis on this topic. It identified 42 unique variables examined as risk factors of SARS-CoV-2 infection. Only two manuscripts were based on real-time infection surveillance programs in community settings during the pandemic.^18^,^32^ In doing so, the need to strengthen collection and reporting of data from communities were highlighted.^1^ Similarly, some of the manuscripts assessed PCR testing records with other multisectoral data to hence presented unique approaches to identify risk factors.^1^,^4^ Both abovementioned trends indicated a modern, more holistic, and multidisciplinary approach towards global health scenarios.^21^,^39^ Finally, identified risk factors like lower SES and occupation could encourage quality prognostic research to update infection prevention and promote overall health literacy.^7^

### Limitations:

Limiting to three bibliographic databases and the period 2023-24 might have resulted in missing out on possibly informative data. The latter was intended to allow a time lag for more representative evidence body considering the capacity constraints of publishing robust health data in several countries. However, the final selection remained dominated by Western European countries (Fig.2). The SARS-CoV-2 pandemic has been reported using a variety of criteria, terminologies, and jargon.^45^ While recently there seemed to be increasing interest in consensus statements, it might have resulted in varying search yields across the three bibliographic database we used.^19^,^28^ Notwithstanding the pandemic, risk factor like SES, occupations, health status, lifestyles, and PCR testing protocols biological specimen collection have varied by sociocultural, professional, and geographical contexts.^2^,^30^,^31^ Therefore, it warranted caution while interpreting our findings because several manuscripts were based on PCR records of 2020-21 when testing guidelines varied regularly worldwide.^10^

Furthermore, individuals below 18 years of age were part of the study samples in 12 manuscripts in small proportions.^2^-^4^,^9^,^10^,^16^,^20^,^22^,^30^,^31^,^33^,^36^ Similarly, one manuscripts also included individuals who were either hospitalized or from hospital staff.^17^ The lack of representation of Asian and African countries also impacted the generalizability of this evidence.

## CONCLUSIONS

Low socioeconomic status and occupation emerged as the most consistent risk factors for SARS-CoV-2 infection, with limited representation of low- and middle-income countries in evidence body. Future research should prioritize standardized methods and inclusion of underrepresented regions to enhance global applicability and inform targeted public health interventions.
